# Microbiome factors in HPV-driven carcinogenesis and cancers

**DOI:** 10.1371/journal.ppat.1008524

**Published:** 2020-06-04

**Authors:** Daniel Lin, Ramez Kouzy, Joseph Abi Jaoude, Sonal S. Noticewala, Andrea Y. Delgado Medrano, Ann H. Klopp, Cullen M. Taniguchi, Lauren E. Colbert

**Affiliations:** Department of Radiation Oncology, Division of Radiation Oncology, University of Texas MD Anderson Cancer Center, Houston, Texas, United States of America; University of Michigan Medical School, UNITED STATES

## Introduction

Human papillomavirus (HPV) infections account for over 600,000 new cancer cases every year [1]. HPV is implicated in approximately 70% of oropharyngeal cancers (OPCs), 90% of anal cancers, and virtually all cases of invasive cervical cancer (ICC) in the U.S [2]. HPV carcinogenesis is mediated by its E6 and E7 oncoproteins, which force differentiating epithelial cells to re-enter the cell cycle to grow and increase viral production [3]. Although most HPV infections resolve over time, persistent infection can cause catastrophic cell-cycle instability and eventually lead to invasive cancer [2]. Nevertheless, HPV presence alone is insufficient for cancer formation. Factors unique to the individual mucosal sites such as epithelial surface integrity, mucosal secretions, immune regulation, and the local microbiota likely play a role in HPV persistence and progression to cancer [2–4]. Dysbiosis of the microbiome can have profound effects on overall health and has recently been linked to cancer progression and treatment responses [5].

Methodological advances in microbiome sequencing and analysis have enabled these recent sweeping advances in knowledge. In particular, 16S ribosomal RNA (rRNA) amplicon sequencing is frequently used. This cost-effective method sequences specific hypervariable regions of the 16S rRNA gene and clusters the identified bacteria into operational taxonomic units (OTUs) that can quantify diversity metrics and relative abundances, as well as provide genus-level identification [6]. However, 16S rRNA subregion sequencing has inherent disadvantages, including the inability to provide species-level identification and metagenomic functionality.

These deficiencies could be overcome with whole-genome shotgun (WGS) sequencing. WGS deciphers broad regions of entire microbial genomes with significantly more sequencing depth but at a greater cost and use of bioinformatics resources than 16S rRNA analysis [7]. Despite these barriers, notable advantages include enhanced species-level identification and accuracy, improved microbial diversity detection, insight into genome functionality and structure, and the ability to identify nonbacterial microorganisms such as viruses and fungi, which are also critical parts of a tissue’s microbiome [7]. Used in conjunction, these complementary methods may provide further clues to understanding the microbiome’s role in HPV carcinogenesis.

HPV cancers may be uniquely affected by the microbiome since these solid tumors arise in the mucosa of the orogenital tract, which each have unique and diverse microbiomes. Insights into the potential influence of the microbiome on viral persistence, immune response, host-mucosal environment, and cancer treatments for HPV-related cancers are just beginning to emerge. In this review, we will discuss how the microbiome may play a pivotal role in the formation of HPV-driven cancers.

## Microbiome factors in cervical intraepithelial neoplasia

The role of the microbiome in HPV-driven diseases has been extensively studied in cervical intraepithelial neoplasia (CIN). Table 1 presents an overview of key CIN and CIN-related (CIN/ICC) microbiome studies. In 1992, one of the first studies characterizing CIN microbiota utilizing laboratory culture discovered the characteristic presence of “abnormal vaginal flora,” which future studies would later confirm through 16S rRNA analysis [8]. Through the generation of reproducible microbiome archetypes, or community-state types (CSTs), 16S sequencing allowed CIN studies to cross-compare findings [9]. The most common CSTs found in CIN patients were CSTs characterized by *Lactobacillus* depletion, anaerobic bacteria predominance, and *Lactobacillus iners* dominance. These CSTs were significantly associated with preinvasive disease, increased disease severity, and disease invasiveness. Additionally, increased vaginal microbe diversity and richness were associated with increased rates of HPV infection and persistence, as well as higher CIN severity [10–13]. *Sneathia* was significantly enriched in CIN samples in multiple studies, and its presence was associated with changes in immune mediators [12,14]. But, similar to the majority of “enriched microbiota” found using 16S, its pathogenic or protective role is not well understood.

**Table 1:** Summary of articles discussing the microbiome and HPV carcinogenesis.

	Author	Year	Study Subjects	Study Region	Microbial Analysis	Sample Type	Key Bacterial Organisms	Main Finding
CIN	Guijon [8]	1992	CIN positive (*n* = 106) Control (*n* = 79)	Canada	Culture, Gram staining	cervical (swab)	*Mycoplasma hominis*	HPV, abnormal vaginal flora, and *M*. *hominis* were all significantly associated with CIN.
	Piyathilake [11]	2016	CIN3 (*n* = 132) CIN2 (*n* = 208) CIN1 (*n* = 90)	United States	V4 16S rRNA	cervical (sponge)	*L*. *iners*	Alpha and beta diversity was not significantly associated with disease status. *L*. *iners* dominance was associated with increased disease severity.
	Klein [13]	2019	LSIL (*n* = 72) HSIL (*n* = 50) Control (*n* = 23)	Tanzania	V4 16S rRNA	cervical (brush)	Mycoplasmatales, Pseudomonadales, Fusobacteria, *Staphylococcus*	Increased alpha diversity was associated with HSIL and HPV. Brush samples from HSIL patients revealed unique associations with Mycoplasmatales, Pseudomonadales, and *Staphylococcus*.
CIN/ICC	Mitra [10]	2015	LSIL (*n* = 52) HSIL (*n* = 92) ICC (*n* = 5) Control (*n* = 20)	United Kingdom	V1–V2 16S rRNA	cervical (swab)	*Lactobacillus* spp., *L*. *crispatus*, *Sneathia sanguinegens*	*Lactobacillus* depletion, high diversity, and species richness was associated with increasing disease severity and high-risk HPV positivity.
	Audirac-Chalifour [12]	2016	HPV− control (*n* = 10) HPV+ control (*n* = 10) SIL HPV+ (*n* = 4) ICC HPV+ (*n* = 8)	Mexico	V3–V4 16S rRNA	cervical (swab, biopsy)	*L*. *iners*, *Sneathia* spp., *Fusobacterium* spp.	Increased alpha diversity in ICC and SIL with unique beta diversities at every stage of ICC. All 4 study groups were dominated by a single distinct population of bacteria: *L*. *crispatus*, *L*. *iners*, *Sneathia* spp., and *Fusobacterium* spp. were dominant in. HPV-negative samples, HPV-positive samples, SIL samples, and ICC samples, respectively.
	Łaniewski [14]	2018	Control (*n* = 51) LSIL (*n* = 12) HSIL (*n* = 27) ICC (*n* = 10)	United States	V4 16S rRNA	cervical (swab, lavage)	*Lactobacillus* spp., *Sneathia* spp.	Decreased abundance of *Lactobacillus* spp. and increased microbiome diversity was associated with increasing severity of cervical neoplasm and ICC.
	Kwon [21]	2018	CIN (*n* = 17) ICC (*n* = 12) Control (*n* = 18)	Korea	Whole-genome sequencing	cervical (swab)	*Alkaliphilus*, *Pseudothermotoga*, *Wolbachia*, *Lactobacillus*, *Staphylococcus*, *Candidatus*, *Endolissoclinum*	Diversity was not significantly associated with disease status. ICC and CIN were each significantly enriched with bacteria unique to the other disease status.
ICC	Wang [22]	2019	ICC (*n* = 8) Control (*n* = 5)	China	V4 16S rRNA	gut (fecal)	Proteobacteria, *Parabacteroides*, *Escherichia-Shigella*, *Roseburia*	Increased alpha diversity (NS) and differing beta diversity of gut microbiome in ICC versus control. Seven genera differentiated significantly in relative abundance between ICC and controls.
	Sims [23]	2019	ICC (*n* = 42) Control (*n* = 46)	USA	V4 16S rRNA	gut (fecal)	*Prevotella*, *Porphyromonas*, *Dialister*	Increased alpha diversity and differing beta diversity in ICC versus control. ICC patients exhibited signifcantly enriched *Prevotella*, *Porphyromonas*, and *Dialister* when compared to age, race, and BMI matched controls.
HNSCC	Guerrero-Preston [26, 29]	2016 2019	OPC HPV+ (*n* = 7) OPC HPV− (*n* = 4) OCC HPV− (*n* = 6) Control (*n* = 25)	USA	V3–V5 16S rRNA; high-resolution 16S rRNA analysis	oral (saliva)	*Gemella*, *Leuconostoc*, *Streptococcus*, *Dialister*, *Veillonella*, *L*. *gasseri*/*L*. *johnsonii*, *L*. *vaginalis*, *Fusobacterium*	[26] Decreased richness and diversity seen in OPC and OCC tumor samples versus controls. [29] Longitudinal study patients showed a decrease in alpha diversity following surgery, with an eventual increase for patients that recurred. [29] Species-level resolution revealed an enrichment of *L*. *gasseri*, *L*. *johnsonii*, *L*. *vaginalis*, and *F*. *nucleatum* in tumor samples.
	Lim [28]	2018	OCC/OPC HPV+ (*n* = 31) OCC/OPC HPV− (*n* = 21) Control (*n* = 20) Risk control (*n* = 10)	Australia	16S rRNA	oral (oral rinse)	*Rothia*, *Corynebacterium*, *Paludibacter*, *Porphyromonas*, *Capnocytophaga*, *Haemophilus*, *Gemella*	Decreased alpha diversity in OCC and OPC versus controls. OCC and OPC patients shared unique relative abundance trends in specific bacteria when compared to control samples.

**Abbreviations:** CIN, cervical intraepithelial neoplasia; CST, community-state type; HNSCC, Head Neck Squamous Cell Carcinoma; HPV, human papillomavirus; HSIL, high-grade squamous intraepithelial lesion; ICC, invasive cervical cancer; LSIL, low-grade squamous intraepithelial lesion; NS, not significant; OCC, oral cavity cancer; OPC, oropharyngeal cancer.

*Lactobacillus* species have been better studied and appear to serve primarily protective roles with some exceptions. *L*. *crispatus* dominance has been strongly associated with a healthy vaginal microbiome and is responsible for producing high quantities of lactic acid and secretion of protective proteins throughout mucosal microenvironment [15]. Conversely, *L*. *iners* is the most commonly reported *Lactobacillus*-dominated CST in CIN patients. *L*. *iners* produces low amounts of lactic acid with no reported host-protective peptide production. *L*. *iners* CSTs are also the most likely to transition to CSTs characteristic of CIN patients, possibly because of its ability to adapt to a variety of pH environments and its distinct lack of bacteriocin synthesis genes, all of which allow abnormal cervicovaginal bacteria to thrive [15,16].

An association between bacterial vaginosis (BV) and CIN has long been suggested [17]. CIN and BV present similar vaginal microbiomes characterized by decreased *Lactobacilli* abundance, increased predominance of abnormal anaerobic bacteria, and increased diversity [17]. Studies suggest a variety of mechanisms in which BV may result in HPV persistence and CIN. Decreased abundance of lactic-acid–producing *Lactobacilli* resulting in abnormally high vaginal pH (>4.5) can cause bacterial overgrowth and decreases in protective flora [14]. This disruption in the colonization of protective microbiota can result in weakened defense mechanisms to fend off viral infections. BV is also associated with increased production of epithelial-lining–degrading enzymes which can allow HPV infection to initiate [17]. Additionally, BV has been associated with increased levels of proinflammatory cytokines and chronic inflammation at mucosal sites [18]. Women with BV expressed increased levels of cytokine interleukin (IL)-1β and decreased levels of antiinflammatory molecule SLPI (secretory leukocyte protease inhibitor) [18]. Furthermore, toll-like receptors (TLRs) act as a first-line of defense in recognizing viral infection and foreign bacteria. TLR9 has been suggested to recognize HPV infections and initiate an immune response, while E6 and E7 oncoproteins directly down-regulate TLR9 at a transcriptional level [19]. BV incidence has been associated with SNPs in TLR2/7, but their exact roles in BV are not well understood [20]. All of these bacterial, mucosal, and immune complications related to BV can result in an increased susceptibility to HPV infection and the development of high-grade intraepithelial lesions.

## Microbiome factors in ICC

HPV persistence is necessary but insufficient for the formation of ICC, and it is now believed the local microbiota may play a role in tumorigenesis. Table 1 provides a summary of ICC-related microbiome studies. One study utilized shotgun metagenomic sequencing to reveal potential metabolic and functional roles of the microbiome involved in inflammation and defense mechanism pathways but additional WGS studies are required to confirm these findings [21]. Additionally, the gut microbiome and its role in ICC is beginning to emerge. Fecal samples from ICC patients exhibited unique gut microbiota composition with greater diversity when compared to healthy-matched controls [22,23]. Gut dysbiosis has been associated with tumorigenesis through inflammation and cytokine modulation, but its role in HPV clearance and cervical carcinogenesis is still unclear [5]. Our group’s ongoing studies suggest that both the cervical and gut microbiome are associated with treatment response in cervical cancers [24].

16S rRNA studies assessing the cervicovaginal microbiome account for the majority of ICC studies. ICC patients exhibit decreases in abundance of *Lactobacillus* spp., increases in overall bacterial diversity and richness, and an increased predominance of *Fusobacterium* spp. As with CIN, the ICC microbial profile resembles that of BV. The transition of CIN to invasive disease does not seem to result in major vaginal microbiome shifts, as similar states of dysbiosis facilitate the persistence and progression of HPV-driven disease. Although observed in both cervical disease states, *Fusobacterium* predominance was more commonly observed in ICC patients. *Fusobacterium* was found to be associated with increased levels of IL-4 and transforming growth factor (TGF)-β1 mRNA, suggesting a role in immunosuppression within the ICC microenvironment [12]. Overall, 16S rRNA analysis demonstrates similar shifts within the ICC and CIN microbiome profiles that likely begin with the disappearance of *Lactobacillus* spp. This decline in the predominating protective bacteria results in increased vaginal pH levels, weakened pathogenic defenses, and damaged mucosal barriers [14,17,25]. Eventually, these conditions allow for opportunistic anaerobic and microaerophilic bacteria to thrive driving the diversity of the cervical microbiome to a state of dysbiosis. Foreign bacteria cause disrupted immune responses and elevated inflammation levels [20]. Together, these factors contribute to an optimal environment for HPV carcinogenesis.

## Microbiome factors in other HPV-driven cancers

Despite numerous studies assessing the microbiome in oral cavity cancers (OCCs) and OPCs, most of these studies do not specify HPV positivity or instead focus on non-HPV–related oral cancers. The section labeled HNSCC (Head Neck Squamous Cell Carcinoma) in Table 1 presents the studies focusing on HPV-positive oral cancers and OPCs. In unknown HPV-status OCCs, bacteria colonizing the tumor site were determined to be significantly different than matching contralateral normal mucosa samples [26]. Furthermore, sensitive microbial variations at intraoral sites like the tooth surface, gums, and tongue exist [27]. These findings suggest the importance of site sampling and sampling collection methods when comparing oral microbiome analyses. When considering HPV positivity, HPV-positive OCC and OPC patients exhibit distinct oral microbiome profiles from both healthy controls and HPV-negative OCC and OPC patients, suggesting the presence of HPV influences the composition of the oral microbiome [26,28]. HPV-positive OCC and OPC patients both showed an abundance of *Gemella* and *Leuconostoc*, while *Haemophilus* correlated with HPV infection [26,28]. 16S rRNA sequencing on saliva and oral rinse samples of OCC and OPC patients revealed a decrease in richness and diversity when compared to control patients [26,28]. This decrease in diversity is opposite to cervical patients and suggests that a few dominating, pathogenic bacteria may be influencing HPV persistence and carcinogenesis in the oral environment.

Interestingly, *Lactobacillus* spp. were found to be significantly associated with HPV-positive OPC patient saliva samples [26,29]. In a follow-up study, species-level context was provided for the *Lactobacillus* spp. using high-resolution 16S rRNA analysis. A subset of OPC patient samples were enriched with commensal species from the vaginal flora, including *L*. *gasseri*/*johnsonii* and *L*. *vaginalis*, not seen in control groups nor saliva from the Human Microbiome Project [29]. The discovery of *Lactobacillus* in the oral microbiome of these samples is not well understood, as *Lactobacillus* is often protective in vaginal and oral contexts [29]. It was suggested that these normally commensal vaginal species could have been transferred to the oral flora during oral sex, which, if validated, would have interesting implications in the role of vaginal-associated *Lactobacillus* during oral HPV disease.

Beyond cervical cancers and OPCs, research characterizing the microbiome of other HPV-driven cancers is relatively nonexistent. Characterization of the anal cancer microbiome would be particularly interesting because of direct interactions with the gut microbiota, but studies have yet to be published, likely because of disease rarity.

## Conclusion and future directions

The identification of bacterial composition changes in HPV-associated cervical cancers and OPCs has provided valuable insight into potential target populations of interest and possible biomarkers of disease and disease-state.

CIN and ICC patient 16S rRNA bacterial profiles have largely been established across multiple studies, and they exhibit shared characteristics pathognomonic to BV, including decreased *Lactobacillus* presence, increased microbial diversity, and increased predominance of atypical, anaerobic bacteria. These distinct features of BV are involved in the overall weakening of the immune and mucosal defense response against invading pathogens and pathogen clearance through a variety of mechanisms, including the release of mucin-degrading enzymes, disrupted pH maintenance, cytokine modulation, and chronic inflammation. In comparison, research characterizing the microbiome of HPV-driven oral and oropharyngeal cancers is limited. But still, microbiome profiles of OCC and OPC patients exhibit significantly different microbiota compositions when compared to healthy controls. Additionally, these cancers exhibit a significant decrease in microbial diversity, which will likely benefit future studies because the pool of potentially pathogenic bacteria is much more limited. Specific genera found within the oral microbiome of HPV+ OPC and OCC patients like *Gemella*, *Leuconostoc*, and, unexpectedly, commensal vagina *Lactobacillus* spp. provide an interesting starting point for future investigations.

The microbiome field regarding HPV-driven cancers is quickly emerging with the majority of research dedicated to characterizing bacterial profiles, but much is still unknown. Outside of HPV-driven cancers, microbiome research on viruses, fungi, and other microorganisms of the human flora and their relationships to disease are gaining interest [30,31]. Bacteria have also been reported to affect cancer treatment response to chemotherapy and immunotherapy in both preclinical and clinical studies in non-viral–related cancers [32,33]. To our knowledge, preclinical animal studies investigating the HPV microbiome have not been performed, possibly because of suboptimal papillomavirus-tumor models and difficulties in modeling the human microbiome in animals. Furthermore, the majority of HPV-related carcinoma studies utilize cross-sectional sampling methods. The addition of longitudinal studies would provide unique information into changes within the microbiome throughout disease progression, treatment, and follow-up.

While all HPV-driven cancer microbiomes require additional taxonomic characterization, the field would benefit immensely from mechanistic studies that describe the relationship of microbiota and the immune response to HPV infection, clearance, and carcinogenesis. More sensitive sequencing techniques and metabolomics could provide insight into specific microorganisms and associated gene expression and metabolite changes. Combining these data with rigorous immune analyses such as antigen stimulation, immune profiling, and computational analysis of sequence homology could provide critical molecular clues in understanding the microbiome’s role in tumor immunity activation and anergy. Continued understanding of the pathogenic microbiota through clinical investigation and validation in preclinical models could help rapidly shed light on this evolving field.
